# Mutation Status and Epithelial Differentiation Stratify Recurrence Risk in Chordoid Meningioma—A Multicenter Study with High Prognostic Relevance

**DOI:** 10.3390/cancers12010225

**Published:** 2020-01-17

**Authors:** Maria-Magdalena Georgescu, Anil Nanda, Yan Li, Bret C. Mobley, Phyllis L. Faust, Jack M. Raisanen, Adriana Olar

**Affiliations:** 1 Department of Pathology, Louisiana State University, Shreveport, LA 71103, USA; yli2@lsuhsc.edu; 2 Feist-Weiller Cancer Center, Shreveport, LA 71103, USA; 3 NeuroMarkers Professional Limited Liability Company, Houston, TX 77025, USA; 4 Department of Neurosurgery, Rutgers University, Camden, NJ 08901, USA; an651@rwjms.rutgers.edu; 5 Department of Pathology, Vanderbilt University Medical Center, Nashville, TN 37232, USA; bret.mobley@vumc.org; 6 Department of Pathology and Cell Biology, Columbia University, New York, NY 10032, USA; plf3@cumc.columbia.edu; 7 Department of Pathology, the University of Texas Southwestern Medical Center, Dallas, TX 75390, USA; jack.raisanen@utsouthwestern.edu; 8 Department of Pathology and Laboratory Medicine and Neurosurgery, Medical University of South Carolina and Hollings Cancer Center, Charleston, SC 29425, USA; adriana_olar@yahoo.com

**Keywords:** chordoid meningioma, epithelial differentiation, NHERF1/EBP50, NF2, TRAF7, ATM, chromatin remodeling genes

## Abstract

Chordoid meningioma is a rare WHO grade II histologic variant. Its molecular alterations or their impact on patient risk stratification have not been fully explored. We performed a multicenter, clinical, histological, and genomic analysis of chordoid meningiomas from 30 patients (34 tumors), representing the largest integrated study to date. By NHERF1 microlumen immunohistochemical detection, three epithelial differentiation (ED) groups emerged: #1/fibroblastic-like, #2/epithelial-poorly-differentiated and #3/epithelial-well-differentiated. These ED groups correlated with tumor location and genetic profiling, with *NF2* and chromatin remodeling gene mutations clustering in ED group #2, and *TRAF7* mutations segregating in ED group #3. Mutations in *LRP1B* were found in the largest number of cases (36%) across ED groups #2 and #3. Pathogenic *ATM* and *VHL* germline mutations occurred in ED group #3 patients, conferring an aggressive or benign course, respectively. The recurrence rate significantly correlated with mutations in *NF2*, as single gene, and with mutations in chromatin remodeling and DNA damage response genes, as groups. The recurrence rate was very high in ED group #2, moderate in ED group #3, and absent in ED group #1. This study proposes guidelines for tumor recurrence risk stratification and practical considerations for patient management.

## 1. Introduction

Meningiomas are heterogenous tumors and their pathologic classification in the WHO Classification of Tumors of the CNS comprises 13 recognized histologic variants [1]. Moreover, meningiomas are segregated based on numerous different histologic criteria into three grades of increasing aggressiveness, WHO I to III, with higher grade correlating with increased potential for recurrence. These criteria include mitotic rate, histologic variant, brain invasion, necrosis, increased cellularity, sheeting, prominent nucleoli and decreased nuclear-cytoplasmic ratio [1]. This rather complicated histologic classification does not always accurately predict tumor recurrence, and recent efforts have attempted to more precisely stratify meningiomas using genetic profiling. In this respect, mutations in *TERT* promoter have been found to be linked to worse prognosis [2,3]. Mutations have been found to correlate with tumor location and histologic variant: mutations of *TRAF7*, *KLF4*, *SMO*, *POLR2A*, and *AKT1* have been found to correlate with a skull base location [4,5,6] whereas mutations of *SMARCE1*, *BAP1* or a combination of *TRAF7* and *KLF4* mutations have been shown to be associated with clear cell, rhabdoid or secretory variants, respectively [5,7,8,9].

The most common mutations found in meningioma involve the *NF2* gene, with up to 60% of tumors demonstrating somatic inactivation of *NF2* [10]. *NF2* mutations are found in both sporadic meningiomas, with a predilection for the fibrous and transitional variants preferentially located at the convexity [11], and in the meningiomas occurring as part of the NF2 syndrome [12].

Chordoid meningioma is one of the four more aggressive histologic variants, considered to be a WHO grade II neoplasm [1]. Diagnosis of this variant is based on morphology alone. Molecular profiling of chordoid meningiomas has not been performed, mainly due to the paucity of cases. By assembling a representative cohort of cases, we have previously shown that this variant is characterized by epithelial differentiation (ED) with microlumen formation [13]. We have also found that immunohistochemistry (IHC) for NHERF1, an adaptor protein that interacts with the ezrin-radixin-moesin (ERM)-NF2 family of proteins to structure microvilli [14,15], specifically highlights microlumens [13,16]. In the current study, we analyzed a 30-patient cohort, performing mutational profiling by next generation sequencing (NGS) and correlating the findings with ED, age, gender, location, and recurrence rate. The combined data resulted in delineation of the mutational landscape of chordoid meningioma and stratification of the patients at risk for recurrence, with important implications for clinical management.

## 2. Results

### 2.1. NHERF1 IHC Defines Three Chordoid Meningioma ED Groups that Correlate with Tumor Location

**Figure 1:**
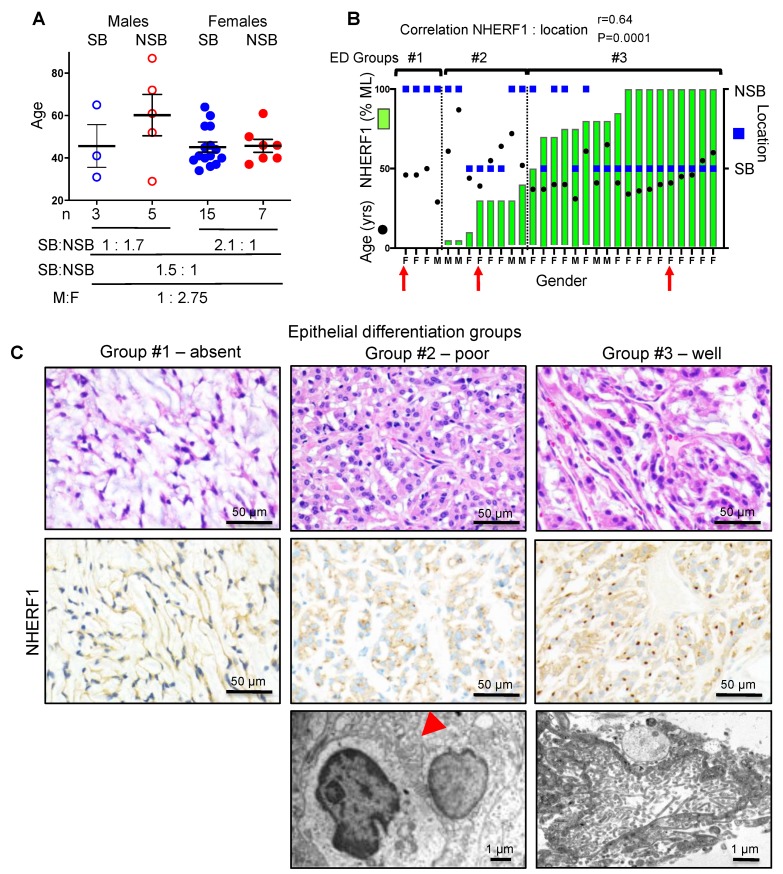
NHERF1 microlumen (ML) extent reveals three epithelial differentiation (ED) groups in chordoid meningioma. (**A**) Age, sex, and location for a cohort of 30 patients with chordoid meningioma. The individual distribution and mean ± SEM are indicated. M, male; F, female; SB, skull base; NSB, non-skull base. (**B**) Graphic representation of NHERF1 ML distribution, age, and location reveals three ED groups and significant correlation of NHERF1 ML extent with SB location: r, Spearman coefficient. (**C**) H&E, NHERF1 IHC, and electron microscopy images from the 3 representative cases in each ED group, indicated with red arrow in (**B**). Red arrowhead indicates a pocket with microvilli.

**Figure 2:**
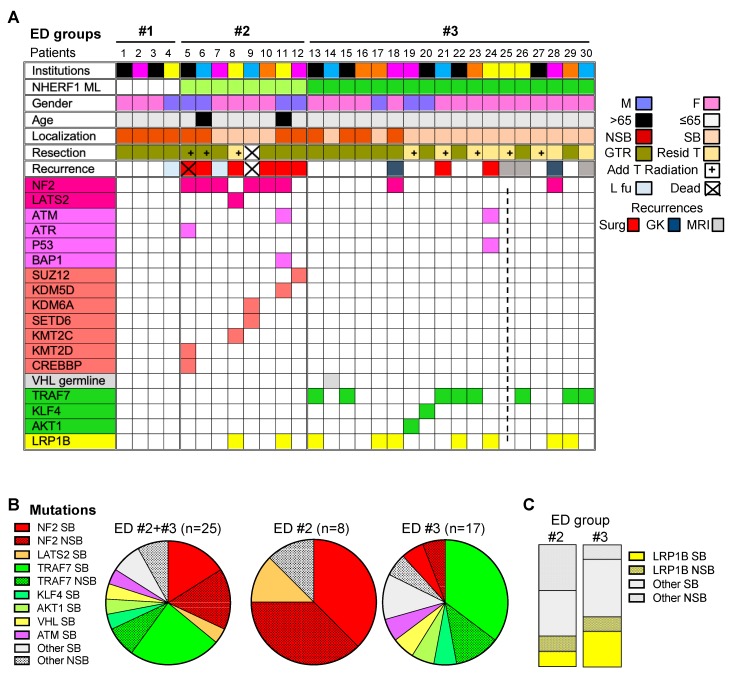
NHERF1 ED groups show distinct gene mutation profiles. (**A**) Comprehensive color-coded table in which columns correspond to individual patients/tumors ordered as in Figure 1B. Samples originating from the same institution have the same color. The genes within the same pathway are color-coded, except for TRAF7, KLF4, and AKT1, that are grouped together because of their frequent occurrence in SB tumors. The dotted line indicates the intraosseous tumor for which NGS could not be performed. M, male; F, female; GTR, gross total resection; Resid T, residual tumor; Add T radiation, additional tumor radiotherapy; L fu, lost to follow-up; Surg, surgery; GK, gamma knife; MRI, regrowth on MRI without any treatment. (**B**) Pie charts indicating the correspondence between the most prevalent gene mutations and tumor location. (**C**) Stack distribution of *LRP1B* mutations in tumors from ED groups #2 and #3.

A cohort of 30 patients with chordoid meningioma, WHO grade II, was assembled from multiple medical centers (Figure 1 and Figure 2, and Table 1). The female to male to ratio was 2.75:1 (Figure 1A), which is comparable to the gender ratio of meningioma in the general meningioma population [1]. The median age at onset was 44.5 years old, which is significantly lower than the median age of 65 years old in the general population [1]. Although there was a nine-year difference between the mean age of women (45.32) and men (54.75), this difference was not statistically significant (Figure 1A). Sixty percent (n = 18) of the tumors were located at the skull base (SB) (Figure 1A and Table 1), in contrast to previous studies where the majority of tumors were non-skull base (NSB) [1,17]. However, NSB tumors were predominant in men, with a SB:NSB ratio of 1:1.7, while the SB tumors were predominant in the female population, with a SB:NSB ratio of 2.1:1 (Figure 1A). Left side laterality was seen more frequently (17 patients) than right side (11 patients) (Table 1).

We have previously shown that chordoid meningioma is characterized by ED with formation of microlumens reliably detected by NHERF1 IHC [13]. We scored the extent of NHERF1 microlumen formation in the tumors and found significant correlation between the extent of microlumen labeling and tumor location (*p* = 0.0001) (Figure 1B and Table S1). We also found the extent of microlumen formation to segregate the tumors into three ED groups: (1) tumors with fibroblastic appearance and absent NHERF1 microlumen staining found only in a NSB location, predominantly in women (F:M = 3:1), (2) tumors with epithelial morphology showing small NHERF1 dots in less than 50% of the tumor, equally occurring in male and female patients in NSB and SB locations, and (3) tumors with epithelial morphology showing diffuse and robust NHERF1 microlumen staining, located predominantly at the SB (SB:NSB = 3.5:1) and occurring mainly in women (F:M = 5:1) (Figure 1B,C). Electron microscopy was performed on tumors with epithelial differentiation from the last two groups (Figure 1C). It showed pocket-like small extracellular spaces with irregular microvillus-like projections in a poorly differentiated chordoid meningioma from the 2nd group, and well-formed microlumens in well-differentiated tumors from the 3rd group, in good correlation with the histology and NHERF1 immunostaining.

### 2.2. Chordoid Meningioma ED Groups Exhibit Distinct Gene Mutation Profiles

To define the mutational landscape of chordoid meningioma, we were able to perform NGS on 31 tumors from 29 patients, including 27 initial tumors and four recurrences (Figure 2A). For 17 tumors, the initial results were confirmed by NGS on larger gene panels, mainly by whole exome sequencing (15 tumors). Two additional debulking resection specimens were sequenced and had the same mutational profile as the initially resected specimens. The SB tumor from one female patient (F25) was entirely within the bone, and the sample could not be processed for NGS due to prior decalcification (Figure 2A, dotted line).

*NF2* mutations were found in 6 tumors from ED group #2 (75%) and 2 tumors from ED group #3 (11.8%), significantly correlated with the age of the patient (*p* = 0.0005; r = 0.6), and were equally distributed between SB and NSB tumors (Figure 2A,B, Table 1 and Table S1). All NF2 mutations were non-syndromic. The SB location corresponded to the anterior and middle cranial fossae in one and three cases, respectively, in contrast to the predominant posterior fossa location reported for *NF2*-mutated meningiomas, in general [5]. All mutations resulted in protein truncation, either by frameshift, nonsense mutation or splice site alteration, and were accompanied by loss of heterozygosity (LOH) (Table S2). A *LATS2* in-frame deletion insertion missense variant, SL425PP, was detected in the SB tumor from F8 that did not harbor *NF2* or chromosome 22 alterations (Figure 2A,B, Tables S2 and S3), raising the possibility of independent mutations in the NF2-Hippo pathway in this ED group. Interestingly, in the F8 tumor, the transcriptome analysis showed an aggressive signature, with *EGFR*, *HRAS*, *CCND1*, and *MET* RNA overexpression, although the chromosomal array showed only few alterations in this tumor that did not involve any of the above loci (Table S3).

Heterozygous copy number loss mapping to chromosome 22 *NF2* locus was noted in the absence of *NF2* mutations in one and four tested cases of ED groups #1 and #3, respectively, whereas the two cases without *NF2* mutations from ED group #2 had intact *NF2* locus (Table 1).

*TRAF7* mutations were detected in 8 tumors from ED group #3 (47%) without correlation with patient’s age but with correlation with NHERF1 microlumen extent, and occurred mainly in SB location (Figure 2A,B, Table 1 and Table S1). Unlike *NF2*, TRAF7 mutations were missense (Q384E, N520S, Q539H, G560C, Y563C, S629T, R653Q), most having been described in meningioma, in correlation with SB location [5,18]. Mutations in other known genes involved in meningioma pathogenesis such as *AKT1* (E17K) and *KLF4* (P238S) were marginally involved in one midline SB tumor, each from ED group #3 (Figure 2A,B and Table S2). The KLF4 P238S mutation from the M20 foramen magnum tumor was distinct from the usual hotspot K409Q mutation reported in meningioma [5], and was previously seen in diffuse large B cell lymphoma [19]. One patient from this group (F14) without either *TRAF7*, *KLF4*, or *AKT1* mutations harbored a germline *VHL* mutation with *VHL* copy number loss in the tumor, and developed posterior fossa chordoid meningioma followed by hemangioblastoma 12 years later. Together, the mutations in *TRAF7*, *KLF4*, *AKT1*, and *VHL* genes were mutually exclusive and occurred in 64.7% of the well-differentiated chordoid meningiomas from ED group #3 (Figure 2A,B, Table S1).

Mutations in *LRP1B*, a gene previously described as mutated in high-grade meningiomas [20], were detected in nine chordoid meningiomas, and occurred in tumors from both ED group #2 (25%) and ED group #3 (41%), without apparent predilection for location (Figure 2A,C). *LRP1B* encodes a large low-density lipoprotein (LDL) receptor family member with tumor suppressor activity [21]. Initially detected as a target for deletion and epigenetic silencing in urothelial and esophageal carcinomas [22,23], missense point mutations spanning the whole gene are commonly found in lung adenocarcinoma [24]. We detected *LRP1B* frameshift, splice and missense variants of unknown significance, some of which having been previously reported in carcinomas or hemangioblastoma [25,26] (Table S2).

Pathogenic alterations in genes involved in chromatin remodeling were detected in 5 aggressive tumors from ED group #2 (62.5%) (Figure 2A, Table 1 and Table S2). Protein-truncating mutations were found in *KMT2D* and *CREBBP* (patient M5), *KMT2C* (patient F8), *KDM6A* and *SETD6* (patient F9) and *SUZ12* (patient M12), and homozygous deletion, in *KDM5D* (patient M11). Mutations in genes involved in DNA damage response (DDR) were found in three aggressive tumors from both ED groups: one *ATR* protein-truncating mutation in the tumor from patient M5, an *ATM* splice mutation and a *BAP1* missense mutation (T613M) previously reported in carcinomas, including prostate carcinoma [27], in the tumor from patient M11, and a pathogenic heterozygous germline *ATM* in-frame deletion insertion mutation, p.D2625_A2626delinsEP (7 providers in ClinVar; ataxia-telangiectasia syndrome and hereditary cancer predisposing syndrome), together with a somatic *TP53* truncating mutation, in the recurrent tumor from patient F24 (Figure 2A and Table S2). With the exception of the F24 tumor, all these mutations clustered in ED group #2, similarly to the *NF2* mutations, and significantly correlated with the recurrence rate (Table S1).

No pathogenic mutations were found in known genes involved in sporadic or familial meningioma *SMO*, *POLR2A*, *SMARCB1*, *SMARCE1*, *PIK3CA*, *PIK3R1*, *AKT3*, *PTEN*, *MEN1*, and *PRKAR1A* [4,5,6,8,9].

### 2.3. Recurrence-Free Survival (RFS) Correlates with ED Groups and Mutation Profiles.

Follow-up information was available for 28 patients (Figure 2A). One patient in each ED group #1 and #2 was lost to follow-up after surgery. Two patients from ED group #2 succumbed, one after developing multiple recurrences (M5), and the other after resection of an aggressive SB tumor (F9). Initial gross total resection was performed for 69% of the tumors, with rates of 100%, 85.7%, and 55.5% for ED groups #1, #2, and #3, respectively (Table 1). Gross total resection was achieved in all NSB tumors and half of SB tumors (Figure 2A), and correlated with tumor location (*p* = 0.0014) and not with recurrence (Table 1). Surgery for recurrence was performed in eight patients, representing 27% and 31% from the entire or adjusted (see below) cohort, respectively, of which six (75%) clustered in ED group #2, and the remaining two (25%), in ED group #3 (Figure 2A). There was no recurrence or death for the patients belonging to ED group #1, whereas all the seven patients with follow-up from ED group #2 had an adverse event (Table 1). Eight SB tumors from ED group #3 did not undergo gross total resection, and five of these received post-operatory fractionated radiation or gamma-knife therapy (Table 1 and Figure 2A). Two of these, F21 and F24, underwent re-resection for recurrence (Figure 2A). Regrowth of residual tumor on MRI, either as 2–3 mm per year or nodular recurrences in the resection bed, were observed in one radiation-treated and four untreated tumors, the latter including two tumors with assumed gross total resection (Figure 2A). Three radiation-treated residual tumors did not show regrowth. Regrowth was significant for two tumors, F18 and F28, that were gamma-knife treated (Figure 2A). After adding the five tumors with MRI regrowth to the two surgical recurrences, the cumulated surgical and radiologic recurrence for ED group #3 was 39% (Table 1).

**Figure 3:**
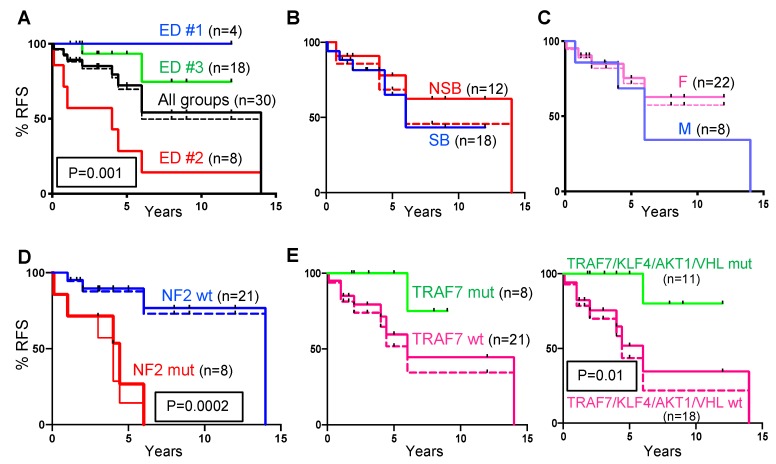
Recurrence-free survival (RFS). RFS color-coded Kaplan–Meier curves are shown with *p*-values when statistical significance is achieved. The number of patients corresponding to each group is shown in brackets. Color-coded dotted lines correspond to adjusted RFS curves after the removal of the four ED group #1 cases. (**A**) Significantly decreased surgical RFS in ED group #2 compared to group #3. (**B**,**C**) No significant difference for location and sex (M, males; F, females). (**D**) Significantly decreased RFS in the *NF2*-mutated (mut) compared to *NF2*-wild-type (wt) groups. The thin and thick red lines correspond to all-treated (surgery and gamma-knife) and surgery-treated *NF2*-mut cases, respectively. Statistical-significant differences: *NF2*-mut all-treated vs. *NF*2-wt (*p* = 0.0002, shown) and adjusted (*p* = 0.0006); *NF2*-mut surgery-treated vs. *NF*2-wt (*p* = 0.0028) and adjusted (*p* = 0.0067). (**E**) Weak protective effect of *TRAF7* mutations (left), with statistical difference when combined with *KLF4*, *AKT1* and *VHL* mutations (right).

For surgically-resected recurrences, the median RFS was 14 years for the entire cohort, four years for ED group #2, and undefined for ED groups #1 and #3 (Figure 3A). All three ED groups appeared to exhibit distinct RFS, with statistically significant differences between ED groups #2 and #3 (*p* = 0.001) (Figure 3A). As the ED group #1 showed a distinct overall clinic-pathologic profile, with NSB location, absence of NHERF1 microlumens, and lack of overall recurrence, an interpretation of metaplastic meningioma, WHO grade I, appears to correspond more accurately to the biology of these tumors. Therefore, we adjusted the RFS of the entire cohort by removing the four patients from ED group #1 (Figure 3A, black dotted line). Consequently, the median RFS for the adjusted chordoid meningioma cohort dropped from 14 to 6 years (Figure 3A, dotted line). The RFS at five years was 72.3% for the entire cohort, 70% for the adjusted cohort, and 14.3% and 93.3% for ED groups #2 and #3, respectively (Figure 3A).

The RFS was also compared between SB and NSB tumors (Figure 3B). In contrast to other variants of meningioma, where an SB location confers worse RFS [28], a similar trend of RFS was noted in both locations for chordoid meningiomas, with or without adjustment for ED group #1 NSB tumors. A similar overlapping RFS profile without significant differences was noted for male and female patients (Figure 3C).

*NF2* mutations in chordoid meningioma strongly and significantly lowered patient RFS (Figure 3D). All the patients with *NF2*-mutated tumors underwent surgical resection or gamma-knife therapy for recurrence within the first 6 years after the initial surgery. The median RFS was 4 and 4.42 years for all-treated and only surgery-treated *NF2*-mutated patients, respectively (Figure 3D, thin and thick red lines, respectively) in comparison to 14 years for adjusted or unadjusted *NF2*-wild-type patients. From all the mutations, the mutations in *NF2* as single gene were the only ones showing significant correlation with the recurrence rate (*p* = 0.0001/r = 0.63 and *p* = 0.018/r = 0.43 for all-treated and surgery-treated recurrences, respectively), whereas as gene groups, chromatin remodeling genes and DDR genes showed also significant correlations with the recurrence rate, especially for surgery-treated recurrences (*p* = 0.00003/r = 0.68 and *p* = 0.004/r = 0.5, respectively) (Table S1). Conversely, *TRAF7* mutations showed an opposite trend without reaching statistical significance, with one patient having a *TRAF7*-mutated tumor undergoing surgery for recurrence (F21) (Figure 2A and Table S1). The median RFS in the *TRAF7*-mutated group was undefined, whereas in the *TRAF7*-wild-type group was six years, without reaching statistical significance between groups (Figure 3E, left. A combined *TRAF7/KLF4/AKT1/VHL*-mutated profile showed statistical significance with (*p* = 0.009) or without (*p* = 0.028) adjustment for ED group #1 (Figure 3E).

### 2.4. DDR Germline or Somatic Mutations Induce Aggressive Tumor Phenotypes in Chordoid Meningioma

In our cohort, three patients had pathogenic DDR gene mutations and all had an aggressive course of disease (Figure 2A). Patient M5 developed multiple tumors following the first resection and succumbed eight years later due to disease. His initial poorly differentiated ED group #2 tumor was sequenced and showed an aggressive signature with pathogenic mutations in *NF2*, *ATR*, *CREBBP* and *KMT2D*. A nonsense mutation in RET receptor tyrosine kinase at Y1090* was also detected. Since the patient died, we did not have matched normal DNA control for comparison, and therefore we do not know if one of these mutations was in the germline. However, the patient had subsequent resections with high grade tumors lacking chordoid histology (not shown). Patient M11 presented with a chordoid NSB tumor with very high mitotic activity that harbored *NF2* nonsense mutation with LOH, *PTEN* homozygous deletion, *ATM* splice variant and *BAP1* missense mutation. He developed a recurrence less than one year later, which additionally showed *KDM5D* homozygous deletion, and was subsequently placed in hospice. The recurrent tumor harbored chordoid morphology but with a decrease in NHERF1 microlumen extent from 30% to 5% [13]. It is noteworthy that *BAP1* germline and somatic mutations have been linked to an aggressive course in high grade rhabdoid meningiomas [8].

**Figure 4:**
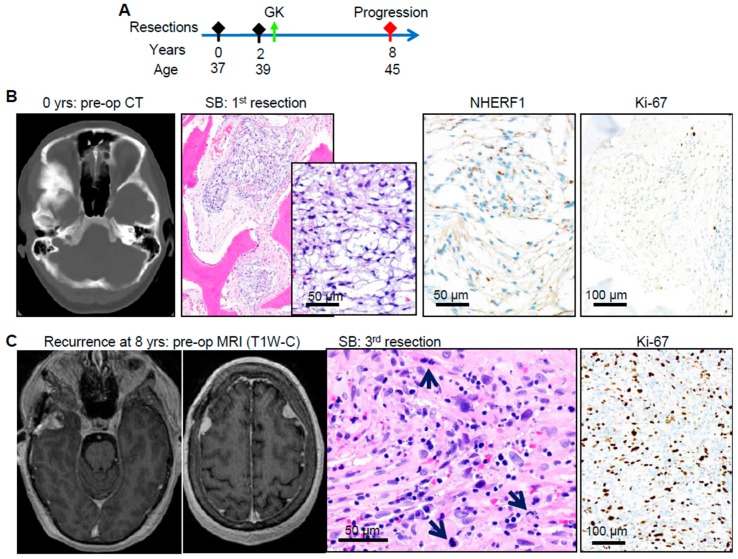

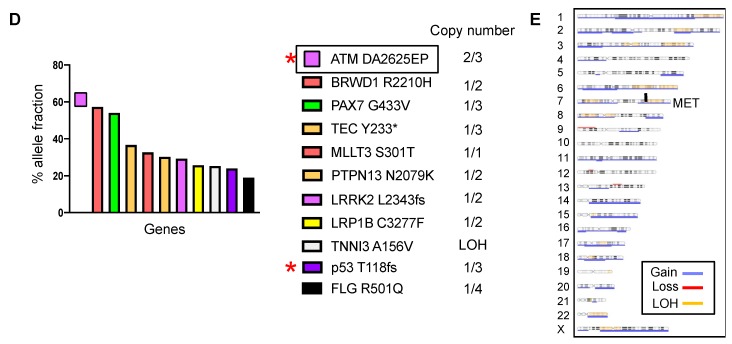
Aggressive course of chordoid meningioma in a young patient with *ATM* heterozygous germline mutation. (**A**) Time-course of disease. GK, gamma knife radiation. (**B**) Initial tumor: pre-operatory (pre-op) CT and H&E preparation show chordoid meningioma within the bone. NHERF1 IHC reveals microlumens and Ki-67 proliferation is low. (**C**) Re-recurrence: MRI T1W sequence with contrast shows multiple tumors, including re-recurrence of the initial SB tumor. H&E shows sarcomatous transformation with multiple mitotic figures (black arrows) and Ki-67 IHC reveals a high proliferation index. (**D**) Mutation profile of the re-recurrence showing germline *ATM* mutation (boxed) with copy number gain and miscellaneous somatic mutations, including a pathogenic (red asterisk) *TP53* mutation. The copy number for each gene was compiled from the allelic fraction and the chromosomal array. (**E**) Chromosomal array showing multiple gains, LOH regions, and only few losses. The positioning of *MET*, the only gene showing RNA overexpression is indicated.

A third patient, F24, had an unexpectedly aggressive course for an initially well-differentiated, mitotically quiescent, ED group #3 SB chordoid meningioma in a young woman. Local recurrence developed after two years, and re-developed despite surgery and gamma-knife therapy after six years together with three other bilateral NSB tumors (Figure 4A,C). Initially diagnosed as chordoid meningioma at the 1st resection, with diffuse expression of NHERF1 microlumens and a low Ki-67 proliferation index (Figure 4B), the 3rd resection showed sarcomatous transformation of the recurrent tumor, absence of NHERF1 microlumens, and high mitotic and Ki-67 proliferation indices (Figure 4C). Because of specimen decalcification, the 3rd resection specimen was the only tumor amenable to sequencing. Matched tumor/normal NGS identified a germline pathogenic *ATM* mutation with copy number gain in the tumor (Figure 4D). Ten somatic mutations were identified, of which the *TP53* truncation mutation was annotated as pathogenic, and the rest as variants of unknown significance. Of these, a deleterious truncating mutation (L2343fs) was noted in LRRK2, a protein recently described as an ATM substrate with a role in regulating p53 in response to genotoxic stress [29]. This finding suggests that the DDR in this tumor was altered by multiple hits in the ATM/p53 axis. Similar to the *RET* Y1090* truncating mutation from the M5 tumor, a truncating mutation in another tyrosine kinase, *TEC* Y233*, was present in this tumor as a variant of unknown significance. The other mutations were missense, and included two chromatin remodeling genes, *BRWD1* and *MLLT3*, and also *LRP1B*. The chromosomal microarray in this tumor showed a complex pattern with multiple chromosomal gains, LOH and only few losses, including a heterozygous loss of *CDKN2A/B* locus (Figure 4E and Table S3). Although the *EGFR* and *CCND1* (cyclin D1) loci were triploid, and the *MET* locus was triploid with LOH, the transcriptome analysis showed only *MET* RNA overexpression in this tumor, contrasting with the findings from F8 (Table S3). 

## 3. Discussion

**Figure 5:**
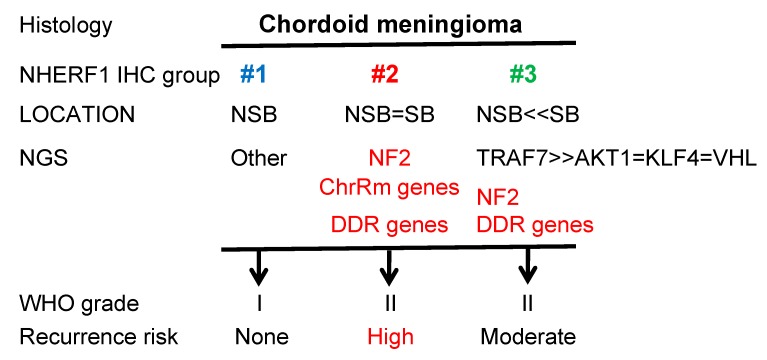
Flow chart illustrating the step-wise analysis of chordoid meningioma and the proposed grading depending on the recurrence risk. ChrRm, chromatin remodeling; DDR, DNA damage response.

Chordoid meningioma is a rare, aggressive variant of meningioma for which correlated clinical-histological-NGS studies have not been performed due to paucity of cases. We have performed an integrated study of the largest chordoid meningioma cohort to date, finding three groups of chordoid meningiomas with sharp recurrence risk stratification that can be readily distinguished by conventional clinico-pathologic investigations. We therefore propose stepwise practical recommendations for neuropathologists and neurosurgeons (Figure 5). Noteworthy overall demographic characteristics different from previous reports [1] are the younger age of onset and the SB location. Regarding tumor location, our study contrasts with the initial largest clinic-histologic study performed by Couce et al. where a NSB tumor predominance was reported [17]. In that study, only 34 cases had over 50% chordoid histology and would fit the diagnostic criteria for WHO grade II chordoid meningioma, however statistical analysis was performed on a total of 42 cases. In addition, that study may have involved surgical bias as its cases were derived from a single institution, whereas our multicenter study included cases from five US institutions. Further, our cases were evenly represented among the ED groups and genetic profiles (see Figure 2A), and therefore our work is likely to reflect more accurately the overall distribution of chordoid meningioma.

By using the clinically-validated NHERF1/EBP50 as marker for microlumens [13,16,30], chordoid meningiomas were divided into three ED tumor groups (Figure 5). Whereas the very rare meningiomas from ED group #1 may be reclassified as WHO grade I, the tumors from the other two groups warrant a higher WHO grade. In particular, the ED group #2 tumors that present with pathogenic mutations in *NF2* and/or chromatin remodeling genes confer high risk of recurrence, and it is our recommendation to grade them at least as WHO grade II, high risk. A moderate risk for surgical recurrence with insidious growth of many of the residual SB-located tumors, even if greatly improved compared to the study reported 19 years ago by Couce et al. [17], are hallmarks of ED group #3, and therefore a denomination of WHO grade II, moderate risk, may be applied to these. In patients with aggressive course, suspicion for possible germline mutations in DDR genes should be raised. *TRAF7* and *NF2* mutations had the same incidence, each occurring in roughly one third of the adjusted cohort, and were mutually exclusive, as previously noted [5]. We therefore recommend subjecting all chordoid meningiomas for NGS analysis with panels containing *NF2*, chromatin remodeling genes, DDR genes, and if possible, *TRAF7*. Since some of these tumors may be mainly intraosseous, a critical point is to alert the neurosurgeon and train pathology personnel for obtaining material amenable to NGS. An alternate NGS-only approach for chordoid meningioma management in hospital settings lacking clinically-validated NHERF1 IHC may segregate cases into high recurrence risk, when *NF2*, DDR and chromatin remodeling gene mutations are present (Figure 5, red labeling), and low recurrence risk, when mutations in other genes are detected (see Table S1). In this approach, NHERF1 IHC may be sent for outside testing to confirm the very rare fibroblastic-like cases from the ED group #1.

The ED-genetic correlations in our cohort are striking. Interestingly, NHERF1 interacts with all the members of the ERM-NF2 family [15,31], although in microlumens from various tumors and in normal ependyma and choroid plexus, it only co-localizes with ezrin and moesin and not with NF2 (merlin) [13,16,32]. In its physiological localization at the plasma membrane, NHERF1 has been shown to act as a tumor growth and invasion suppressor in vitro and in vivo [33,34,35,36]. Moreover, its loss has been shown to impair microvilli formation and lumen morphogenesis [14,37,38]. It is likely that its loss in ED group #2 contributes to the aggressive phenotype of these tumors, although it may not be the determining event. The most common genetic event in ED group #2 was *NF2* gene mutation. Knowing that *NF2* mutations are the most common single gene mutation in meningioma [10] and that multiple meningiomas arise in half of the patients with the NF2 syndrome [39], it is most likely that the *NF2* mutations are the initiating event for the aggressive tumors from ED group #2. Importantly, *NF2* mutations were only detected in the aggressive tumors from our cohort and were significantly correlated to RFS. Interestingly, *NF2* mutations are commonly associated with the low-grade WHO I fibrous and transitional variants, where they are found in 70–80% of the tumors [11,40]. Clues for reconciling the discrepancy between adverse prognosis in chordoid meningioma and lack of adverse prognosis in low-grade meningiomas may come from an in vitro study where the isolation of meningioma cells lines was hampered when the patient tumor lacked NF2 expression [41]. The NF2-deficient meningioma cells exhibited senescence and reduced cell growth in comparison to matched normal arachnoidal cells or to NF2-expressing meningioma cells, and the same phenotype was obtained by silencing NF2 in normal arachnoidal cells [41]. Interestingly, following serial passaging, NF2-deficient but not NF2-expressing meningioma cultures developed foci with aggressive phenotype, manifested by anchorage independence and rapid cell proliferation [41]. Although sequencing was not performed for the foci, it is likely that compensatory mutations developed in other genes. In our aggressive cases, NGS confirmed that the *NF2* alterations co-occurred with deleterious mutations in other genes, including chromatin remodeling and DDR genes, and we hypothesize that the combination of these mutations in the context of NF2 deficiency is responsible for the aggressive phenotype. In particular, we detected pathogenic mutations in an array of chromatin remodeling genes and correlated these to high risk in meningioma.

The detection of a germline pathogenic mutation in *ATM* in a patient from ED group #3 with aggressive disease course is the first evidence that, in addition to other neoplasms [42], heterozygous *ATM* mutations also predispose to meningioma. Although a slight increase of an *ATM* haplotype frequency has been reported in meningioma compared to control population [43], this is the first reported case of meningioma developing in a patient with an *ATM* germline cancer-predisposing mutation. Interestingly, a quasi-triploid/tetraploid genome was detected in this case despite a relatively non-aggressive transcriptome signature. An explanation for this disparity may come from studies showing that ATM inhibition induces tetraploidization of mesenchymal stem cells in order to prevent cell senescence, most likely by checkpoint adaptation [44,45]. Mutations in DDR genes were present in other aggressive tumors, with statistical correlation to recurrence rate.

## 4. Materials and Methods

Human specimens: Formalin-fixed paraffin-embedded (FFPE) meningioma resection specimens were obtained from the authors’ institutions. This study used only cases of chordoid meningioma, WHO grade II, diagnosed if more than 50% of the morphologic pattern was present in the tumor [46] and graded according to the 2016 WHO Classification of Tumors of the CNS [1]. The demographics included patient age and gender, and tumor location. These studies were performed in compliance with the ethical guidelines of the Helsinki Declaration and approved by institutional review board (IRB) committees.

Histology, IHC, and imaging: The specimens were processed for H&E staining and IHC with anti-NHERF1/EBP50 antibody 1:2000 (Thermo/Fisher, Waltham, MA, USA) on a Ventana Benchmark Ultra platform (Roche/Ventana Medical Systems Inc., Tucson, AZ, USA), as described [13]. Images were acquired at various magnifications with a Nikon Eclipse Ci microscope equipped with a Nikon Digital Sight DS-Fi2 camera (Nikon Instruments Inc., Melville, NY, USA), by using the Nikon NIS Elements 4.51.00 program.

Transmission electron microscopy: An approximately 8 mm^3^ tumor sample was processed for electron microscopy examination, as previously described [13]. Ultrathin sections were stained with either 3% uranyl acetate or UranyLess (Electron Microscopy Sciences, Hatfield, PA, USA) and lead citrate and examined in a Hitachi 7650 electron microscope (Hitachi High Technologies, Schaumburg, IL, USA). Digital images were obtained by using AMT Image System (Advanced Microscopy Techniques, Danvers, MA, USA).

NGS: Nucleic acids were extracted from FFPE samples, as previously described [47]. The generation and preparation of the customized 295-gene DNA library based on hybrid-capture/enrichment SureSelect XT HS technology (Agilent, Santa Clara, CA, USA) were described [47]. The FASTQ files were analyzed with the SureCall program (Agilent), as described [47]. For comparison and confirmation, 17 samples from 15 patients were independently sequenced at Tempus Labs (Chicago, IL, USA) by using the xE (whole exome) or xO (1713 genes) libraries. Matched normal samples were available only for the two patients with germline cancer-predisposing mutations, and RNA sequencing and transcriptome analysis was also performed for them (Tempus Labs).

Copy number variation analysis: Single nucleotide polymorphism microarray-based chromosome analysis was performed for two cases using the IScan^®^ System with the CytoSNP-850K v1.1 BeadChip (Illumina, San Diego, CA, USA) and analyzed using GenomeStudio (Illumina) and Nexus, version 9.0 (BioDiscovery, Inc., El Segundo, CA, USA) software, as previously described [47]. Copy number analysis was also performed for an additional 14 cases undergoing whole exome NGS (Tempus Labs).

Statistical analysis: The NHERF1-labeled microlumen density and extent were assessed as previously described [13]. A cut-off value of 50% was established for the extent of microlumens in a representative tumor section, based on morphological differences between tumors with diffuse (≥50%), or patchy/focal (<50%) microlumen extent. Numerical data were examined for normality of distribution and expressed as mean ± SEM, unless mentioned otherwise, by using the GraphPad Prism program (GraphPad Software, La Jolla, CA, USA). Differences between groups were assessed by using unpaired two-tailed t-test with or without Welch’s correction for variances significantly different, and non-parametric data correlation was assessed by Spearman correlation coefficient (r), as described [48,49]. Kaplan-Meier RFS analyses using Log-rank (Mantel-Cox) and Gehan-Breslow-Wilcoxon tests were performed on patients with treated recurrences, as previously described [33]. Statistical significance was considered for *p* < 0.05. Confidence intervals for all tests were 95%.

## 5. Conclusions

In conclusion, we assembled the largest multi-institutional cohort of WHO grade II chordoid meningiomas and conducted an integrated clinic-histologic-genomic analysis. From this analysis, we derived three clinically-relevant groups, recognizable by using routine laboratory tests (Figure 5). We identified *NF2* mutations, chromatin remodeling and DDR gene mutations as independent prognostic markers for aggressive course, and described the first case of meningioma developing in a patient with *ATM* germline cancer-predisposing mutation. We therefore propose considerations for WHO classification, including downgrading ED group #1 tumors to WHO grade I neoplasms, assigning greater risk to ED group #2 tumors, and revising the location for chordoid meningioma. The high clinical relevance of this analysis addresses current clinical needs for a better, field-oriented risk stratification system in meningioma [50].

## Figures and Tables

**Table 1:** Chordoid meningioma: demographic, clinical, histologic and genomic characteristics.

ED Groups	ED #1 n = 4; 13.3%	ED #2 n = 8; 26.7%	ED #3 n = 18; 60%	ED #2 + #3 n = 26; 86.7%	Total n = 30; 100%
Age (median)	46	58	40.5	42.5	44.5
Gender Male:Female	1:3	1:1	1:5	1:2.7	1:2.75
Location SB	0%	50%	78%	69.2%	60%
Laterality Left:Right:Midline	1:1:0	5:3:0	5:3:1	7.5:4.5:1	8.5:5.5:1
Gross total resection	100%	85.7%	55.5%	64%	69%
Radiation post-surgery	0%	43%	28%	32%	27.6%
^1^ Surgical recurrence/death	0%	100%	11.1%	36%	32%
^2^ Additional MRI recurrence	0%	-	27.8%	20%	18%
^3^ Cumulated recurrence/death	0%	100%	38.9%	56%	50%
NHERF1 ML extent (average)	0%	22.5%	87%	67.1%	58%
^4^ Chr 22q12.2 (NF2) CN loss NF2	25% (n = 4) 0%	71.5% (n = 7) 75%	85.7% (n = 7) 11.8%	78.6% (n = 14) 32%	66.7% (n = 18) 27.6%
Chromatin Remodeling genes	0%	62.5%	0%	20%	17.2%
DNA damage response genes	0%	25%	5.9%	12%	10.3%
TRAF7	0%	0%	47%	32%	27.6%
^5^ KLF4/AKT1/VHL	0%	0%	17.6%	12%	10.3%
LRP1B	0%	25%	41.2%	36%	31%

^1^ Post-surgery unfavorable events, including surgically-resected recurrence or death. ^2^ Re-growth at the initial resection bed noted on follow-up MRI. The regrowth of tumors after resection (re-recurrences) are not included. ^3^ Combined surgically-resected and MRI-visualized recurrence. ^4^ Heterozygous loss. CN, copy number. The number of cases on which the CN analysis was performed is shown in brackets. ^5^ One mutation of each.

## Supplementary Materials

The following are available online at https://www.mdpi.com/2072-6694/12/1/225/s1, Table S1: Correlation analysis in chordoid meningioma, Table S2: Mutations in chordoid meningioma, Table S3: SNP-microarray and transcriptomics data.
