# Supplementary material for: Mutation Status and Epithelial Differentiation Stratify Recurrence Risk in Chordoid Meningioma—A Multicenter Study with High Prognostic Relevance
Source: Cancers (Basel). 2020 Jan 17;12(1):225. doi: 10.3390/cancers12010225 (PMC7016786; doi:10.3390/cancers12010225)
Supplement: Supplementary file 1 [file cancers-12-00225-s001.pdf]

Supplemental Table S1: Correlation analysis in chordoid meningioma.

p value	Age	Location	GTR	NHERF1-ML	NF2	TRAF7	TRAF7/KLF4/ AKT1/VHL	DDR	ChR
Age									
Location	0.2415								
Gross total resection	0.2643	0.0014							
NHERF1 ML	0.2213	0.0001	0.001						
NF2	0.0005	0.5169	0.287	0.123					
TRAF7	0.185	0.3286	0.656	0.007	0.048				
TRAF/KLF4/Akt1/VHL	0.2335	0.0669	0.642	0.006	0.01				
DDR genes	0.3409	0.3372	0.93	0.706	0.105	0.286	0.1762		
ChR genes	0.1133	0.3342	0.788	0.074	0.068	0.149	0.0657	0.013	
Recurrence surgical	0.0521	0.7552	0.656	0.15	0.018	0.22	0.0601	0.004	3.0E-05
Recurrence treated	0.0148	0.6562	0.466	0.4535	0.0001	0.104	0.0161	0.015	0.0006

r Spearman coefficient	Age	Location	GTR	NHERF1-ML	NF2	TRAF7	TRAF7/KLF4/ AKT1/VHL	DDR	ChR
Age	1								
Location	0.221	1							
GTR	0.214	0.563	1						
NHERF1 ML	-0.230	-0.643	-0.562	1					
NF2	0.598	0.123	0.204	-0.288	1				
TRAF7	-0.248	-0.184	-0.086	0.482	-0.363	1			
TRAF/KLF4/Akt1/VHL	-0.224	-0.339	-0.09	0.492	-0.459		1		
DDR	0.180	0.181	-0.016	-0.072	0.302	-0.201	-0.254	1	
ChR	0.295	0.183	0.052	-0.331	0.337	-0.269	-0.340	0.447	1
Recurrence	0.358	0.059	-0.086	-0.269	0.428	-0.23	-0.347	0.509	0.683
Recurrence treated	0.44	0.084	-0.14	-0.142	0.636	-0.302	-0.435	0.438	0.587

GTR, gross total resection; ML, microlumen extent; DDR, DNA damage response; ChR, chromatin remodeling.
Statistically significant correlations are indicated in red; indirect correlations are indicated in brown.
Recurrence treated, combined surgical and gamma-knife treated.

Supplemental Table S2. Mutations in chordoid meningioma

Pathway	Gene	Nucleotide	Amino acid	Effect	Other	Patient
Hippo/NF2 pathway						
	NF2	c.114+1G>A		Splice	LOH	M6
	NF2	c.361C>T	Q121*	Stop gain	LOH	M5
	NF2	c.362delA	Q121fs	Frameshift		F10
	NF2	c.383_386delATGA	D128fs	Frameshift	LOH	F7
	NF2	c.732T>A	Y244*	Stop gain	LOH	M11
	NF2	c.949del	E317fs	Frameshift	LOH	F9
	NF2	c.955C>T	Q319*	Stop gain	LOH	F18
	NF2	c.1191del	L398fs	Frameshift	LOH	F28
	LATS2	c.1273_1277delTCCCTins[illegible]	SL425PP	Missense		F8
Chromatin remodeling						
	KMT2C	c.650C>A	S217*	Stop gain		F8
	KMT2D	c.16451delAA	E5484fs	Frameshift		M5
	CREBBP	c. 4208delG	R1403fs	Frameshift		M5
	KDM5D			CN loss		M11
	KDM6A	c.3864_3870del	I1289fs	Frameshift		F9
	SETD6	c.536dup	D180fs	Frameshift		F9
	SUZ12	c.1294-3dup		Splice		M12
DNA damage response						
	ATM	c.3403-4dup		Splice		M11
	ATM	c.7875_7876delinsGC	D2625_A2626delinsEP	Missense	Germline	F24
	ATR	c.6592delC	L2198fs	Frameshift		M5
	BAP1	c.1838C>T	T613M	Missense		M11
	TP53	c.348del	T118fs	Frameshift		F24
	LRRK2	c.7029-8_7029delins(8)	L2343fs	Frameshift		F24
Other						
	PTEN			CN loss		M11
	VHL			CN loss	Germline	F14
	HRAS	c.508A>T	K170*	Stop gain		M6
	RET	c.3270T>A	Y1090*	Stop gain		M5
	TEC	c.699C>A	Y233*	Stop gain		F24
	AKT1	c.49G>A	E17K	Missense		M19
	KLF4	c.712C>T	P238S	Missense		M20
	LRP1B	c.2056C>T	R686W	Missense		M11
	LRP1B	c.2771C>G	A924G	Missense		M17
	LRP1B	c.2785G>T	V929L	Missense		F29
	LRP1B	c.9401A>G	D3134G	Missense		F18
	LRP1B	c.9830G>T	C3277F	Missense		F24

LRP1B	c.11200C>A	Q3734K	Missense	F13
LRP1B	c.12161A>C	E4054A	Missense	F28
LRP1B	c.8675delA	N2892fs	Frameshift	F22
LRP1B	c.13415+5G>A	delT	Splice	F8
TRAF7	c.1042C>G	Q384E	Missense	F15
TRAF7	c.1559A>G	N520S	Missense	F23
TRAF7	c.1559A>G	N520S	Missense	F30
TRAF7	c.1617G>C	Q539H	Missense	F22
TRAF7	c.1678G>T	G560C	Missense	F26
TRAF7	c.1688A>G	Y563C	Missense	F29
TRAF7	c.1886G>C	S629T	Missense	F13
TRAF7	c.1958G>A	R653Q	Missense	F21

Supplemental Table S3. SNP-microarray and transcriptomics data.

CN, copy number
Start and stop positions are given relative to [GRCh37]
LOH, loss of heterozygosity

Patient F8:

Chr:Start-Stop	Cyto-bands	CN	Comments
chr1:1-94,049,237	1p36.33 - p22.1	1	60% deletion 1p
chr1:239,527,477-249,250,621	1q43 - q44	1	60% deletion 1q
chr10:59,800,194-64,805,235	10q21.1 - q21.3	4	60% high copy gain 10q
chr10:64,760,686-78,866,173	10q21.3 - q22.3	2	60% LOH 10
chr10:65,345,229-70,174,707	10q21.3	5	60% high copy gain 10q
chr10:70,589,478-77,486,626	10q21.3 - q22.2	4	60% high copy gain 10q
chr10:77,536,364-78,887,346	10q22.2 - q22.3	5	60% high copy gain 10q
chr10:112,154,110-135,534,747	10q25.2 - q26.3	1	60% deletion 10q
chrX:86,813,848-96,186,615	Xq21.31 - q21.33	3	60% duplication Xq
chrX:96,186,615-100,657,809	Xq21.33 - q22.1	4	60% high copy gain Xq
chrX:100,657,809-108,243,681	Xq22.1 - q22.3	5	60% high copy gain Xq

Transcriptomics:

Overexpression: EGFR, HRAS, CCND1 (Cyclin D1), MET. All these chromosomal loci were normal.

Patient F24:

Chr:Start-Stop	Cyto-bands	CN	Comments*
chr1:1-63,461,381	1p36.33 - p31.3	3	+1
chr1:63,513,116-200,118,439	1p31.3 - q32.1	4	high copy gain 1pq
chr1:199,971,261-249,250,621	1q32.1 - q44	2	LOH 1q
chr1:200,259,607-249,250,621	1q32.1 - q44	3	+1
chr2:1-41,939,950	2p25.3 - p21	4	high copy gain 2p
chr2:42,041,338-58,354,422	2p21 - p16.1	3	duplication 2p
chr2:58,459,232-95,485,567	2p16.1 - q11.1	4	high copy gain 2p
chr2:95,595,941-110,086,491	2q11.1 - q12.3	3	duplication2q
chr2:164,568,301-193,198,573	2q24.3 - q32.3	2	LOH 2q
chr2:164,714,987-192,923,303	2q24.3 - q32.3	4	high copy gain 2q
chr2:192,923,303-243,199,373	2q32.3 - q37.3	3	duplication 2q
chr3:1-42,366,706	3p26.3 - p22.1	4	high copy gain 3p
chr3:42,484,782-198,022,430	3p22.1 - q29	3	+3
chr3:78,662,193-104,839,517	3p12.3 - q13.11	2	LOH 3pq
chr3:145,066,106-183,503,634	3q24 - q27.1	2	LOH 3q
chr4:1-49,051,182	4p16.3 - p11	3	+4p
chr5:30,696,023-38,251,005	5p13.3 - p13.2	3	duplication 5p
chr5:141,668,625-180,915,260	5q31.3 - q35.3	4	high copy gain 5q
chr6:1-8,639,771	6p25.3 - p24.3	3	+6
chr6:8,639,771-67,458,231	6p24.3 - q12	4	high copy gain 6p
chr6:67,458,231-171,115,067	6q12 - q27	3	+6
chr6:93,681,057-128,541,388	6q16.1 - q22.33	2	LOH 6q
chr6:138,825,687-171,115,067	6q23.3 - q27	2	LOH 6q
chr7:1-68,379,401	7p22.3 - q11.22	3	duplication 7pq
chr7:11,381,458-36,915,185	7p21.3 - p14.2	2	LOH 7p
chr7:102,624,192-159,138,663	7q22.1 - q36.3	3	duplication 7q
chr7:113,327,166-145,081,764	7q31.1 - q35	2	LOH 7q
chr8:1-63,154,491	8p23.3 - q12.3	3	duplication 8pq

chr8:6,772,913-32,105,334	8p23.1 - p12	2	LOH 8p
chr8:42,582,583-63,247,352	8p11.21 - q12.3	2	LOH 8q
chr8:115,578,307-146,364,022	8q23.3 - q24.3	4	high copy gain 8q
chr9:1-30,403,716	9p24.3 - p21.1	1	deletion 9p
chr9:70,984,372-104,973,771	9q21.11 - q31.1	3	duplication 9q
chr11:1-32,211,032	11p15.5 - p13	3	+11
chr11:32,211,032-42,026,659	11p13 - p12	4	high copy gain 11p
chr11:42,026,659-135,006,516	11p12 - q25	3	+11
chr12:18,729,266-27,199,180	12p12.3 - p11.23	1	deletion 12p
chr13:19,147,562-53,501,307	13q11 - q14.3	3	duplication 13q
chr13:60,076,715-75,376,016	13q21.2 - q22.1	1	deletion 13q
chr14:19,280,733-107,349,540	14q11.2 - q32.33	4	high copy gain 14q
chr15:22,576,118-102,531,392	15q11.2 - q26.3	4	high copy gain 15q
chr15:22,922,449-29,211,558	15q11.2 - q13.1	2	LOH 15q
chr16:1-14,391,139	16p13.3 - p13.12	4	high copy gain 16p
chr16:14,391,139-67,655,860	16p13.12 - q22.1	3	duplication 16pq
chr16:67,709,524-83,024,058	16q22.1 - q23.3	4	high copy gain 16q
chr17:1-11,712,491	17p13.3 - p12	3	+17
chr17:11,712,491-57,723,132	17p12 - q23.1	4	high copy gain 17pq
chr17:37,329,066-49,867,150	17q12 - q21.33	2	LOH 17q
chr17:57,723,132-81,195,210	17q23.1 - q25.3	3	+17
chr18:1-11,073,732	18p11.32 - p11.21	3	+18
chr18:11,028,129-13,467,275	18p11.21	2	LOH 18p
chr18:11,073,732-47,156,730	18p11.21 - q21.1	4	high copy gain 18pq
chr18:18,826,139-28,954,111	18q11.1 - q12.1	2	LOH 18q
chr18:47,156,730-78,077,248	18q21.1 - q23	3	+18
chr19:50,990,086-55,645,528	19q13.33 - q13.42	2	LOH 19q
chr20:1-20,795,202	20p13 - p11.23	3	duplication 20p
chr20:29,820,178-63,025,520	20q11.21 - q13.33	4	high copy gain 20q
chr21:21,259,299-24,643,432	21q21.1 - q21.2	2	LOH 21q
chr21:24,819,148-32,915,961	21q21.2 - q22.11	3	duplication 21q
chr22:17,012,935-51,304,566	22q11.1 - q13.33	4	high copy gain 22q
chr22:19,578,158-51,304,566	22q11.21 - q13.33	2	LOH 22q
chrX:1-18,569,044 Xp22.33 -	p22.13	3	duplication Xp
chrX:38,257,658-155,270,560	Xp11.4 - q28	4	high copy gain Xpq
chrX:38,445,060-71,849,931	Xp11.4 - q13.2	2	LOH Xpq

*All the abnormalities are present in approximately 20% of cells.

MET (7q31.2) – GAIN/LOH DETECTED
EGFR - (7p11.2) - GAIN DETECTED
Cyclin D1 (CCND1) (11q13.3) - GAIN DETECTED

Transcriptomics:

Overexpression: MET.
